# Studies Concerning Gaseous Gangrene

**Published:** 1917-12

**Authors:** 


***Studies Concerning Gaseous Gangrene.*** By M. WEINBERG and P. SÉGUIN. Trans. and abs. from the *Annales de l'Institut Pasteur*, Sept. 1917 (Tome XXXI, p. 442).

The authors in the present article give a summary of the results thus far obtained from their study of gaseous gangrene since 1914. (An earlier contribution of W.'s is reviewed in this number. See p. 128). They announce the publication of a work which will give these results with more detail.

*The Bacterial Flora of Gaseous Gangrene.* — The authors have studied 91 cases; all but 2 were military. They were from all parts of the front.

No cases were caused by aerobes alone. In 24 cases the anaerobes were unaccompanied by aerobes; in 67, they were found in symbiosis with one or more aerobe. In 37 cases there was but a single anaerobe; in 54, there were more than one.

According to their frequency, the anaerobes take precedence as follows: B. perfringens (aerogenes capsulatus) in 70 cases (77 o/o), B. œdematiens in 31 cases (34 o/o), B. sporogenes in 25 cases (27 o/o), B. fallax in 15 cases (16, 5 o/o), Vibrion septique in 12 cases (13 o/o), B. tetani in 9 cases (10 o/o), B. histolyticus in 8 cases (9 o/o), B. aerofœtidus in 5 cases (5.5 o/o), B. putrificus in 2 cases (2 o/o), B. bifermentans in 2 cases (2 o/o), B. Ghon-Sachs II in one case (1 o/o), and B. tertius in 1 case (1 o/o).

The authors call attention especially to the frequency of four organisms, two of which, B. perfringens (aerogenes capsulatus) and B. sporogenes, were already known at the beginning of the war, and the other two of which, B. œdematiens and B. fallax, have been discovered by the authors during the study here outlined.

All writers now agree that B. perfringens is the organism most commonly associated with the infection. The importance of B. œdematiens (found in a little more than 1/3 of the cases) lies rather in the fact of the seriousness of most of the cases in which it is concerned.

Of the aerobes present, streptococci appeared in about 40 o/o of the cases; it seriously affected the prognosis. Diplococci (enterococci) appeared in 33 o/o of the cases. Staphylococci were somewhat less frequent. Gram-negative bacilli appeared in the following order of frequency, B. proteus, B. pyocyaneus, and B. coli. The authors have also isolated B. subtilis and another organism which they describe as "aerobic, spore-bearing, motile" and easily confounded in a hanging drop with V. septique; and, on stained smears, with B. perfringens and B. œdematiens.

The authors next consider the relative seriousness of these various organisms. They declare that only three appear to play a dangerous rôle in gaseous gangrene : these are the B. perfringens (aerogenes capsulatus), B. œdematiens, and Vibrion septique. These have appeared alone or in symbiosis in the majority of the cases studied. In 35 out of the 37 cases where only one bacillus was isolated, one of these three germs was present. The B. perfringens was present in 29 of these; the B. œdematiens in 5, and the V. septique in 1.

In almost every case these three showed high pathogenic powers, killing the guinea-pig within 24 hours after injection. The fundamental rôle played by the B. perfringens is no longer disputed by anyone. The case of the V. septique is not so clear, but the authors believe it is more frequently present and active than most observers suppose. The authors insist that B. œdematiens is, next to B. tetani, the most toxic of all anaerobes found in wounds. Its toxin inoculated in the veins of a guinea-pig kills the animal in from 24 to 48 hours, and both the culture and the toxin of this organism produces in the guinea-pig the same symptoms encounterde in men with the form of gaseous gangrene in which this organism is habitually found.

As to mortality, B. perfringens appears responsible for 19 deaths, B. œdematiens for 12, and V. septique for 4. The authors also attribute 1 death to B. fallax, 2 deaths to the association of B. perfringens and B. œdematiens, and one death to the association of B. œdematiens, V. septique, and B. fallax.

They find that the B. perfringens is responsible for death in about a fourth of all the cases in which it is present, that the V. septique is responsible for death in about a third of the cases in which it is present, and the B. œdematiens for death in about one half of the cases in which it is present. These results seem to tally with what is known of the toxicity of these three organisms. The authors do not consider the action of any of the other organisms so far noted to be comparable in seriousness to the danger from these three.

*Hemocultures.* — Of the 20 cases in which the B. perfringens was found in the wound, 15 showed the organism in the blood. Hemoculture was practiced before death on 9 patients, and was 4 times positive; practiced after death on 13 cases, it was 11 times positive. The authors think that this organism does not always pass into the blood—perhaps only in the most advanced stages.

They find also that B. œdematiens passes very rarely into the blood before death. None of the other organisms described is found invariably in the blood before or after death. The authors therefore conclude that blood cultures are an unreliable method of diagnosis, and they further believe that death is due rather to systemic intoxication from the organisms than to septicemia. This intoxication is probably complex in most cases, as several highly toxic organisms are often present in the flora.

The authors classify the clinical types of gaseous gangrene as (1) Classic, (2) Toxic, and (3) Mixed.

*Classic Gaseous Gangrene* they characterize as having the following symptoms : " abundant gas production, considerable gaseous crepitation, often superficial, bronze tint on the skin, blebs, and in fatal cases septicemia often setting in a few hours before death. " Of this type of gaseous gangrene the authors believe B. perfringens and V. septique, either singly or in symbiosis, to be the causative agents. Sometimes an extremely toxic organism like the B. œdematiens may be associated with the other agents in this type of the disease.

*Toxic Gaseous Gangrene.* — This type differs from the classic in that progressive, spreading edema masks the infiltration of the tissues with gas, and together with general symptoms of intoxication, constitutes the most apparent outward sign of the infection. There is rarely septicemia, even in fatal cases. So different is this type from the classic, that surgeons tend to associate it rather with streptococcic infections (white erysipelas) than with genuine gaseous infections. The authors consider the B. œdematiens the causative agent of this form of the disease, although they state that the B. perfringens sometimes produces similar results. They give in detail a case in which the B. œdematiens was responsible, and which was cured after it had reached an alarming stage by the use of anti-œdematiens serum. They note, however, that the two organisms are often associated, and that the action of one may disguise the presence and activity of the other.

*Mixed Gaseous Gangrene.* — This form includes cases which present not only the complex flora (B. œdematiens and B. perfringens) but also the leading symptoms of the two preceding varieties (classic and toxic). These symptoms are usually edema and gaseous crepitation. They cite a case in which there was present on the first day only the edema, but which on the second day developed gaseous crepitation, bronze erysipelas, and blebs.

The authors add, however, that not all cases are as simple and clearly marked as the three classes just described. Almost all cases are complicated by the presence of putrefaction due to secondary non-toxic organisms, of which B. sporogenes (which many have confused with V. septique) is the most frequent and most dangerous variety. Many cases, too, are more seriously complicated by the presence of all of the most virulent organisms. The authors note one fatal case in which B. perfringens, B. œdematiens, V. septique, B. fallax, and B. sporogenes were all present.

Passing to a consideration of the manner of the infection, the authors discuss; (1) Mechanical factors; (2) Bacteriological factors.

*Mechanical factors.* — These include traumatism and bony or vascular lesions. So much has been said already on the importance of these factors, and of the action of the projectile in introducing infection from the clothing and soil, that the authors do not wish to treat at length any one of them. They agree that all injured muscle should be excised as early as possible, and they call attention to the great danger attending cases complicated by bone fracture. About three fourths of all fatal cases have been complicated by fracture. They note also the gravity of any interruption of circulation, whether due to the nature of the injury, or to artificial causes. They agree with K. Taylor that gas pressure is an important agent in interrupting the circulation and in producing local asphyxiation. Especially serious is any injury to the great vessels which supply the region of the wound. Even after a considerable lapse of time (often weeks or months) gaseous gangrene has broken out as a result of hindrances to the circulation. They note the occurrence of rare cases in which mechanical action seems to play no part and in which the wound is slight. The onset in such instances is due, the authors believe, to severe contamination or to certain associations of virulent organisms.

*Bacteriological Factors.* — As to *the influence of aerobic organisms* upon the anaerobic bacilli of gaseous gangrene, the authors are not prepared to accept the conclusions of H. Tissier, who contends that B. perfringens and the anaerobic organisms of gaseous gangrene in general are innocuous except when assisted in their development by aerobes. Like Taylor and others they have obtained no significant results in laboratory experiments with combinations of aerobes and anaerobes. They conclude in this particular that aerobes may favor to some extent the growth of anaerobes by absorbing oxygen and detracting the activity of phagocytes. Possibly they may also supply toxic or diastasic secretions which stimulate B. perfringens and B. œdematiens. The authors do not consider that they are in possession of enough evidence as yet, to make any further statement on this topic.

As to *the influence of anaerobic organisms,* however, they are better prepared to draw conclusions. They give briefly the results of various experiments with the guinea-pig in which the B. hystolicus, associated with either B. perfringens or B. œdematiens, appears to favor in the most marked manner, the development of the infection. This action the authors explain by the characteristic effect of the B. hystolicus upon the tissues which it invades. They state that it transforms vascular connective tissues into softened hemorrhagic masses which favor the growth of the other organisms in the same manner as the tissues devitalized by projectiles or other agents.

The B. sporogenes also appears to favor the growth of B. perfringens, but does so by a putrid and gaseous destruction of the tissues. Similarly V. septique and B. perfringens stimulate each other.

The authors insist especially upon the fatal nature of the interaction of B. œdematiens with B. perfringens, for the latter is not only stimulated itself by symbiosis, but causes the B. œdematiens to flourish to such an extent that this organism gains the upper hand, and by means of its superior toxicity, is often responsible for death. Inoculation of the guinea-pig with any of these mixed cultures produces the same results as are observed clinically.

As many experiments of this nature as the supply of animals would permit, have been carried out, and the results are given with considerable detail. As far as it is possible to do so, the authors believe that they have established the exact correspondence of the varieties of gaseous gangrene observed clinically with those produced experimentally by the various mixed cultures.

In conclusion they mention the sera which they have prepared against the three most virulent organisms (B. perfringens, V. septique, and B. œdematiens) and they state that the injection of these separately or mixed, has often given encouraging results as a supplement to surgical treatment. They promise a more extended communication on this subject shortly. They believe also that this mixed serum will prove more valuable by way of prevention. They hope that it may come to be used as a matter of routine like the anti-tetanus serum.

